# Sleep architecture and regulation of male dusky antechinus, an Australian marsupial

**DOI:** 10.1093/sleep/zsac114

**Published:** 2022-05-14

**Authors:** Erika Zaid, Alexei L Vyssotski, John A Lesku

**Affiliations:** School of Agriculture, Biomedicine and Environment, La Trobe University, Melbourne, Australia; Institute of Neuroinformatics, University of Zurich/ETH Zurich, Zurich, Switzerland; School of Agriculture, Biomedicine and Environment, La Trobe University, Melbourne, Australia

**Keywords:** EEG, metatheria, REM sleep, sleep deprivation, sleep homeostasis, slow-wave activity

## Abstract

**Study Objectives:**

In this study, we (1) describe sleep behavior and architecture, and (2) explore how sleep is regulated in dusky antechinus (*Antechinus swainsonii*), a small insectivorous marsupial. Our aim is to provide the first investigation into sleep homeostasis in a marsupial.

**Methods:**

Wild-caught male dusky antechinus (*n* = 4) were individually housed in large indoor cages under a natural photoperiod of 10.5 h light/13.5 h dark. Continuous recordings of EEG, EMG, and tri-axial accelerometry were performed under baseline conditions and following 4-h of extended wakefulness.

**Results:**

Antechinus engage in SWS and REM sleep. Some aspects of these states are mammal-like, including a high amount (23%) of REM sleep, but other features are reminiscent of birds, notably, hundreds of short sleep episodes (SWS mean: 34 s; REM sleep: 10 s). Antechinus are cathemeral and sleep equally during the night and day. Immediately after the sleep deprivation ended, the animals engaged in more SWS, longer SWS episodes, and greater SWS SWA. The animals did not recover lost REM sleep.

**Conclusions:**

Sleep architecture in dusky antechinus was broadly similar to that observed in eutherian and marsupial mammals, but with interesting peculiarities. We also provided the first evidence of SWS homeostasis in a marsupial mammal.

**Statement of Significance**

Few studies exist on sleep, and none on sleep regulation, in marsupials. Here, we describe sleep architecture and homeostasis in a small, Australian marsupial, the dusky antechinus. SWS and REM sleep in antechinus resemble the sleep states of eutherian and marsupial mammals, while other features, such as the presence of hundreds of short sleep bouts, more closely resemble sleep in birds. Antechinus are cathemeral with great between-individual variation in the timing, but not amount, of sleep. Following 4-h of sleep deprivation, antechinus engaged in more SWS, longer SWS episodes, and greater SWS SWA; however, they did not show any REM sleep rebound. Future studies should investigate whether REM sleep increases following longer periods of sleep loss in marsupials.

## Introduction

Sleep is an essential animal behavior characterized by restfulness and reduced responsiveness. All animals studied thus far, from mammals and other vertebrates to the simplest invertebrates, have been shown to spend part (or even most) of their life asleep [1–4]. This grand evolutionary persistence suggests that sleep serves an important physiological function, or perhaps different functions in different species [5, 6].

Comparative studies can be a powerful approach to first capture the diversity of sleep behavior and physiology across animals, and then relate that diversity to sleep function. For instance, mammals and birds have two kinds of sleep, slow-wave sleep (SWS) and rapid-eye movement (REM) sleep. Although much of what is known about sleep has been gleaned from the study of rodents and primates [1, 7, 8], studies of other mammals and birds have revealed striking differences in sleep. For example, ruminants and other large mammals can sleep while standing [9–11], marine mammals can sleep while swimming [12, 13], some seabirds can sleep while flying [14], and monotremes and ostriches exhibit a mixed SWS-REM sleep state [15–18]. In this way, the study of “nontraditional” animals has resulted in new insight into conserved, and evolutionarily derived, features and phenotypes of sleep [19, 20].

One prominent feature of sleep thought to be tied to its function is sleep homeostasis. The daily amount of SWS and REM sleep is regulated, such that prolonged wakefulness results in an animal sleeping more, and, in the case of mammalian and avian SWS, sleeping more intensely. The intensity of SWS is measured by the incidence and/or amplitude of slow electroencephalogram (EEG) waves, and quantified as EEG power density below 4.5 Hz, called slow-wave activity (SWA). Under baseline (undisturbed) conditions, both nocturnal (e.g. rat [*Rattus norvegicus*], hamster [*Mesocricetus auratus*]) [7, 21, 22] and diurnal (e.g. chipmunk [*Eutamias sibiricus*], humans) [23, 24] mammals show high SWA at the beginning of the main sleep period, which declines as sleep need dissipates. Conversely, in species with no preference to sleep during the day or night, such as the rabbit (*Oryctolagus cuniculus*) [25], guinea pig (*Cavia porcellus*) [26], and blind mole rat (*Spalax ehrenbergi*) [27], the decline of SWS-related SWA is less clear. Nonetheless, following experimental increases in the duration of wakefulness, SWA is highest at sleep onset and declines as SWA-rich SWS accrues. Such SWS homeostasis has been demonstrated in eutherian mammals, including mice (*Mus musculus*) [28], rats [29, 30], cats (*Felis catus domesticus*) [31–33], ferrets (*Mustela putorius furo*) [34], rabbits [25], tree shrews (*Tupaia belangeri*) [35], and squirrel monkeys (*Saimiri sciureus*) [36]. While eutherian-like SWS homeostasis has also been shown in birds [14, 37–43], no studies have examined sleep regulation in marsupials or monotremes.

REM sleep homeostasis has also been demonstrated in many [25, 44–46], but not all [47], eutherian mammals and birds [37, 38, 48, 49]. Following sleep loss, the amount of REM sleep has been shown to increase while the animal recovers REM sleep lost during the deprivation procedure. However, unlike SWS, there does not appear to be an intensity dimension to REM sleep [7].

To the best of our knowledge, no study has yet investigated sleep homeostasis in marsupials. Some (but few) studies have investigated (1) the behavior and/or electrophysiology of SWS and REM sleep in marsupials, notably the red kangaroo (*Megaleia rufa*) [50], common opossum (*Didelphis marsupialis*) [51, 52], little water opossum (*Lutreolina crassicaudata*) [53], and brushtail possum (*Trichosurus vulpecula*) [54]; (2) the influence of food distribution on sleep and circadian rhythm of the long-nosed potoroo (*Potorous tridactylus*) [55]; and (3) the ontogenesis of sleep states in the North American opossum (*D*. *virginiana*) [56]. Here, we provide the first data on sleep behavior and physiology in antechinus, and we manipulate prior sleep/wake history to explore how SWS and REM sleep are regulated in these Australian marsupials.

## Methods

### Animals and housing conditions

Four male dusky antechinus (*Antechinus swainsonii*) were wild-caught at two different localities in the Otway National Park in south-east Australia (38°45′S, 143°32′E) in March 2019 and transported to La Trobe University in Melbourne. All animals were approximately 6 months old. Age was estimated based on the life-history of antechinus, in which males have a lifespan of 11 months and die at the end of an intense breeding season towards the end of August; conversely, some females produce one litter every year until the age of 2 or 3. Mean body mass at the time of study was 67.3 ± 2.8 g.

Animals were housed individually in rectangular enclosures (129 × 85 × 43 cm high) with a mesh-covered roof to allow illumination and ventilation, and were elevated 1 m off the ground. The floor of each enclosure was covered with sawdust, leaf litter, and natural debris, and enriched with a wooden nest box (30 × 19 × 22 cm high) along with dry eucalypt leaves as bedding material. Structural complexity was added using thick tree branches and bark to create a more natural environment. Each enclosure was equipped with two infrared video cameras, one facing the nestbox and the other inside the nestbox. Clean water was provided *ad libitum* and food (Wombaroo Small Carnivore Mix, Wombaroo Food Products, Australia) was prepared daily. The quantity of food provided was based on each individual’s body mass and adjusted to ensure that excess food was always available. Live mealworms and crickets were provided every second day for additional enrichment. Feeding and spot cleaning were performed in the morning between 0900 h and 1000 h. Antechinus were maintained on a natural photoperiod with lights-on at 0700 h and off at 1730 h; a window on one side of the room provided natural light (sunrise at 0705 h; sunset at 1725 h). During the daylight hours, a quiet recording of native forest sounds was played to provide acoustic enrichment. The temperature in the room was set at 18 ± 5°C, similar to the outdoor temperature at the time of the experiment.

### Electrodes implantation

We characterized sleep and wake using a combination of behavior (via video recordings) and physiology (electrographically). To record the latter, we performed a surgery using standard stereotaxic techniques to implant epidurally-seated cortical electrodes. Briefly, each antechinus was placed on a temperature-controlled heating pad set at 36°C. The animal was anesthetized with isoflurane administered first in an induction chamber and then via facemask (4% for induction; 2% for maintenance in 1 LPM O_2_). The cranium, exposed by a midline incision (*circa* 1 cm length), was cleaned and dried using 3% hydrogen peroxide. Seven holes were drilled through the exposed cranium to the level of the dura. Four holes (0.45 mm diameter) were positioned symmetrically over the left and right hemisphere for recording the electroencephalogram (EEG). In three of the four animals, the anterior row was 2.5 mm caudal to bregma and the posterior row was 2.0 mm rostral to lambda; owing to space constraints arising from a more pronounced temporalis muscle in one individual, the anterior row was 1.0 mm rostral to bregma and the posterior row was 3.0 mm rostral to lambda. These four holes were 1.2 mm, laterally, on either side of the midline. An additional hole was drilled over the right hemisphere for the ground. All electrodes were made of gold-plated, round-tipped pins connected to medical-grade electrode wire (AS633, Cooner Wire, USA). One stainless steel wire electrode was laid upon the nuchal (neck) muscle for the electromyogram (EMG), referenced to the posterior electrode over the right hemisphere. Two larger holes (1.6 mm diameter) were drilled near the center of the rectangular arrangement of EEG electrodes into which stainless-steel bone anchor screws were inserted. All six wires were held in place with a small amount of super glue and soldered to a small (6 mm) connector fixed to the top of the head with dental acrylic (Paladur, Heraeus Kulzer, Germany). The incision site was then closed (to the extent possible) and secured using non-toxic skin glue (Histoacryl, B. Braun, Germany). Post-operative pain and inflammation were managed with meloxicam (0.2 mg/kg, s.c.) administered before the surgery. Animals were given at least 8 days to recover from surgery prior to attaching a “dummy” logger to the headplug. The nonfunctional dummy logger matched the size and mass of the real data logger, which was 2.6 g (less than 5% of total body mass).

All procedures were approved by the: (1) La Trobe University Animal Ethics Committee (AEC 18067), (2) Department of Environment, Land, Water and Planning, and (3) National Parks (permit no. 10007988).

### Experimental procedure

A survey of wild populations of antechinus suggests that they are cathemeral. Specifically, a multi-year trapping study found that dusky antechinus were equally likely to be captured during the day as during the night, suggesting that the animals can be active around-the-clock [57]. To determine the most appropriate time (if any) for a sleep deprivation, we recorded baseline patterns after one-week of habituation to the dummy logger. In doing so, we identified a great deal of between-individual variation in the timing of sleep and wakefulness (e.g., Supplementary Figure S1). Owing to this large variation, we opted for a daytime sleep deprivation between 1200 and 1600 h. The full experiment commenced at least two weeks after the dummy logger had been in place.

It is noteworthy that owing to the light weight of antechinus, we had to use the lightest battery available for the head-mounted data logger (Neurologger 2A, Evolocus, Tarrytown, NY, USA; www.vyssotski.ch/neurologger2.html). The data loggers were powered by two 100 mAh zinc-air batteries (P10, Power One), which enabled a maximum recording duration of *circa* 27 h. Consequently, with a 48-h experimental design, we had to retrieve the data logger, substitute the real logger for a dummy, download the data, and re-attach the original logger with new batteries to each animal halfway through the study. This meant we had an unavoidable 1.5 h gap in our recordings between 1130 and 1300 h. Therefore, the EEG/EMG recordings were obtained during two consecutive 22.5-h periods starting at 1300 h. The first 22.5-h served as a baseline. On the second day, the animals were kept awake from 1200 to 1600 h via a combination of acoustic stimuli, providing live food, and gentle handling. At the end of this 4-h protocol, the antechinus were left undisturbed until the end of the experiment at 1130 h the following day. Note that animals were made accustomed to daily handling, at the correct times, one week prior to starting the experiment so that handling during the experiment would not be unusual.

### EEG recordings and sleep scoring

EEG/EMG recordings were made by the Neurologger 2A. The Neurologger has an inbuilt tri-axial accelerometer to record head accelerations in three dimensions. All bioelectric signals were sampled at 100 Hz. Recordings were manually scored by the same observer (E.Z.) using RemLogic (RemLogic v. 3.4.4, Natus Neuro, USA). Brain state (wake, SWS, REM sleep) was determined for each 4-s epoch using the EEG, EMG, and accelerometry data with the aid of video recordings. Wakefulness was characterized by coordinated body movements, elevated muscle tone, and, when free from movement-related artefacts, relatively low-amplitude, high-frequency EEG activity. Epochs were scored as SWS when more than half of an epoch showed low-frequency activity with an amplitude at least twice that of quiet wakefulness; the emergence of SWS was typically preceded by behavioral signs of sleep, such as immobility and a curled sleep posture, and associated stable, reduced muscle tone. REM sleep was characterized by wake-like EEG activity, but often occurring with reduced muscle tone from the preceding SWS level.

### Spectral and statistical analyses

Fast Fourier Transforms were performed on all 4-s epochs of the right hemisphere EEG data to calculate SWS SWA (0.78–4.30 Hz) and spectral power density for quiet wakefulness, SWS, and REM sleep in 0.39 Hz bins between 0.78 and 25 Hz. Epochs containing artefacts, and transitional epochs, were visually detected and excluded from all spectral analyses. Artefacts were few in the sleep EEG data, typically limited to a twitch or subtle movement causing a solitary high-voltage spike. Conversely, artefacts were prevalent during wakefulness. Periods of active wakefulness were characterized by modulating accelerometer signals, supported by high and variable muscle tone, and the EEG showed blasts of high-voltage activity. Consequently, in awake antechinus, 90% of the EEG signal was not suitable for spectral analysis (range 82%–95%). The 5%–18% remnant of clean signal constituted quiet wakefulness arising from pauses in locomotion or immediately prior to sleep onset.

SWA and spectral power density were analyzed by averaging the 4-s epochs over 1.5-h and 4.5-h time bins, respectively. Power was expressed as a percentage of the 22.5-h baseline SWS mean (for SWA) and as the SWS mean per frequency bin (for spectral power density). All statistical analyses were performed in R Statistical Environment (R Development Core Team 2013) using either one-way analysis of variance (ANOVA) followed by post hoc *t*-tests, or paired *t*-tests, as specified below.

## Results

Few recordings of sleep behavior and physiology have been made on marsupials [1]. Therefore, we begin by describing sleep states in antechinus under baseline conditions. We then quantify the efficacy of our sleep deprivation protocol, and detail the response of the animals. Specifically, we report on the amount of each state, the duration and number of state bouts, changes in SWS-related SWA, and comment on spectral power density patterns.

### Sleep architecture

**Figure 1.**
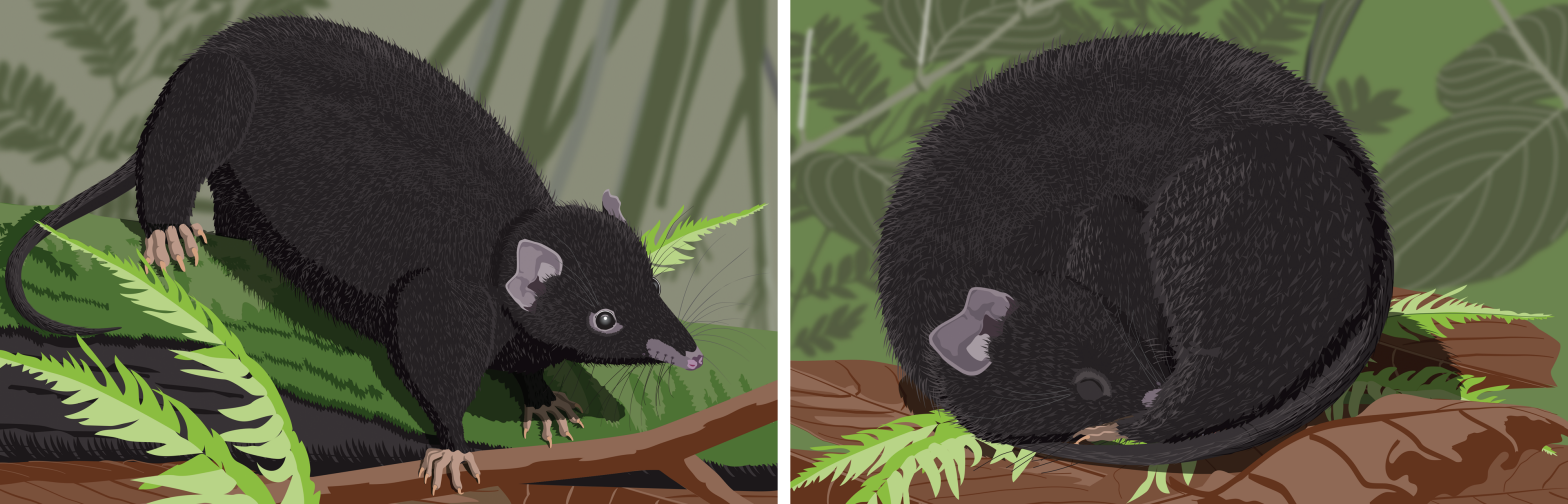
**State-dependent postures in dusky antechinus.** Typical exploratory behavior during wakefulness (left) and species-specific sleep posture (right). In captivity, sleep occurred either in a leaf-litter nest they made themselves, or in the nestbox provided. Prior to falling asleep, each animal would organize the leaves and curl into a ball hiding the head under the body, or with only the nose hidden (behind the leg) and the closed eye visible. In general, the tail followed the round shape of the body and the tip was tucked under the head. Artwork by Damond Kyllo.

**Figure 2.**
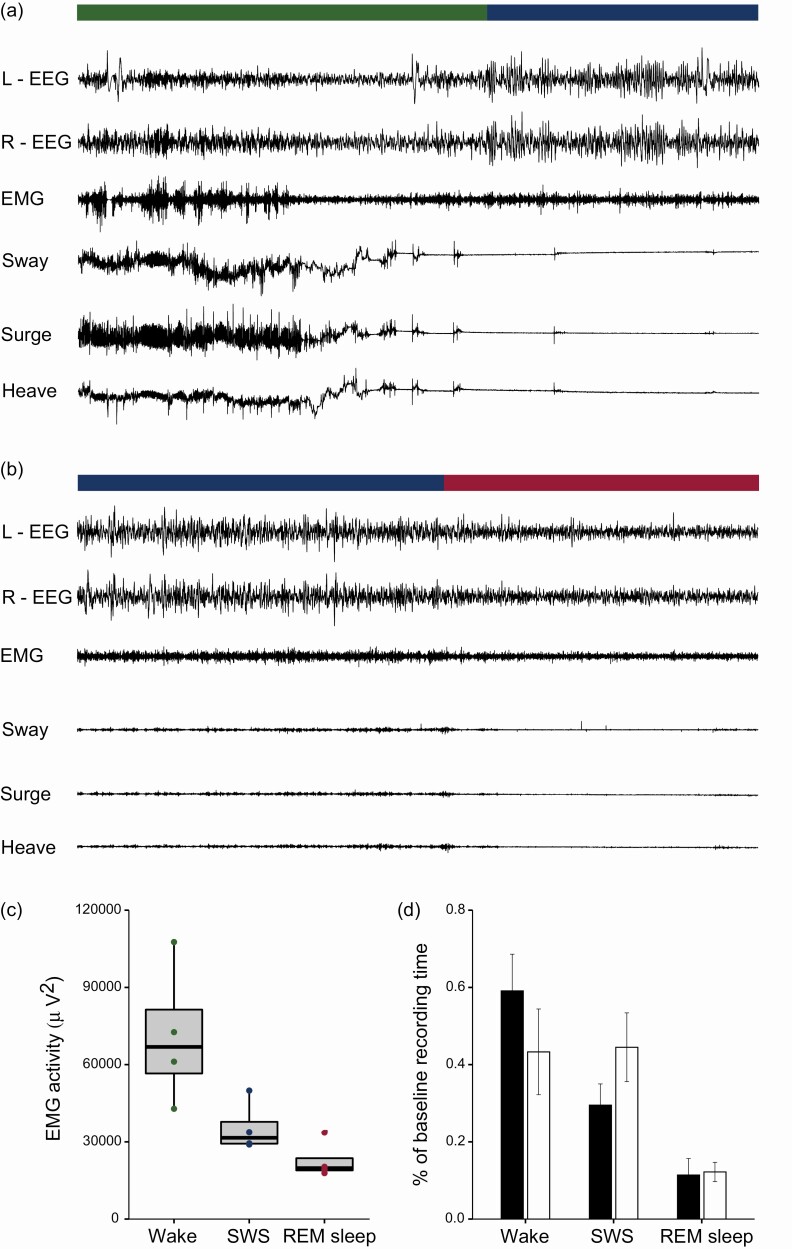
**Sleep physiology in dusky antechinus.** Representative traces showing brain activity (left [L] and right [R] hemisphere electroencephalogram, EEG), muscle tone (electromyogram, EMG), and head movements (accelerometer, ACC) along the three cardinal axes of sway (lateral axis), surge (anterior-posterior), and heave (dorso-ventral) during wakefulness (green bar), SWS (blue bar), and REM sleep (red bar). Wakefulness is characterized by small, fast brain waves, high and variable muscle tone accompanied by large head movements. In (a), the antechinus spent 15 s in quiet wakefulness before entering SWS, characterized by large, slow-waves, low muscle tone, and behavioral restfulness. In (b) during SWS the ACC showed shivering-like activity that ceased upon entering REM sleep. During REM sleep, brain activity resembled wakefulness, but with a further reduction in muscle tone, and an absence of gross body movements. The EEG was filtered using a high-pass filter set at 1 Hz and a low-pass filter of 25 Hz; the EMG was high-pass filtered at 10 Hz and a low-pass filter of 100 Hz; ACC was unfiltered; trace duration is 90 s. (c) Muscle tone varied by state with the highest tone during wakefulness, and the lowest tone during REM sleep. Tone was calculated as the sum of spectral power density between 9.8 and 50 Hz. The boxplots show the median (band inside the box), the upper and lower quartiles (top and bottom edge of each box, respectively), and dots represent individual animals. Letters represent significant differences. (d) The amount of each state during the 22.5-h baseline recording expressed as a percentage of total recording time during the night (black) and day (white); data presented as mean ± SE. For color, please refer to the online images.

Similar to other mammals, awake antechinus were typically characterized by opened eyes and a high amount of activity as animals walked, jumped, climbed, groomed, and fed, as observed in the video and accelerometry recordings (Figure 1). This exertion was supported by the highest and most variable muscle tone (Figure 2a). During such times, the EEG was often contaminated by high neck muscle activity; however, when the animal stopped moving, the EEG was characterized by low voltage, high frequency waves. Prior to sleep onset, antechinus would carefully organize leaves in the nesting site, and groom. They would then assume a curled-up posture, tucking the head under the body. After a short period of immobility (ap. 15 s), the animals would enter SWS. During SWS, the animal could be motionless or show shivering-like activity, as reflected in the accelerometer. Shivering-like activity occurred during the day and night, in all four antechinus, and during most, but not all, SWS episodes. In either case, the EEG was characterized by high amplitude slow-waves and sleep spindles (7–13 Hz). SWS would most often give way to REM sleep, characterized by wake-like brain waves, neck muscle hypotonia, intermittent twitching (visible only on the ear and leg), and a cessation of shivering-like movements (Figure 2b). Indeed, shivering did not occur during any REM sleep episode longer than 4 s. Muscle tone varied by state (*F* = 8.39, df = 2.9, *p* = .009) with the highest tone during wakefulness, and the lowest tone during REM sleep (Figure 2c); EMG activity during REM sleep was lower than that during SWS (*t* = −6.55, df = 3, *p* = .007) and wakefulness (*t* = −3.53, df = 3, *p* = .039).

Under baseline conditions antechinus spent 47% of the 22.5-h day asleep, of which 77% and 23% was SWS and REM sleep, respectively. The sleep pattern appeared to be cathemeral in that there was no significant difference between night and day in the proportion of time spent in each state, nor in the number and duration of state bouts (Figure 2d, Table 1). We observed great between-individual variation in the timing, but not amount, of sleep and wakefulness (Supplementary Figure S1).

**Table 1.** The amount (%), and number and duration (s) of state bouts awake, in SWS and REM sleep under baseline conditions (*mean* ± *SE*). *p*-Values reflect statistical contrasts between night and day.

		Night (13.5 h)	Day (9 h)	Total (22.5 h)	*P*-value
	Wake	59.1 ± 9.5	43.3 ± 11.1	52.8 ± 1.9	0.495
% time	SWS	29.5 ± 5.5	44.5 ± 8.9	35.5 ± 1.8	0.358
	REM sleep	11.4 ± 4.3	12.2 ± 2.5	11.7 ± 1.9	0.919
	Wake	19 ± 5	23 ± 5	20 ± 4	0.601
No. of bouts	SWS	54 ± 15	71 ± 16	61 ± 10	0.534
	REM sleep	39 ± 13	54 ± 13	45 ± 8	0.561
	Wake	904 ± 499	174 ± 54	612 ± 288	0.263
Bout duration (s)	SWS	31 ± 10	39 ± 12	34 ± 11	0.144
	REM sleep	9 ± 3	11 ± 2	10 ± 1	0.644

SWS and REM sleep episodes were short (34 ± 10 s and 10 ± 1 s, respectively). Nonetheless, sleep bouts could last upwards of 10.3 (±3.2) min for SWS and 3.9 (±0.5) min for REM sleep. Long REM sleep bouts occurred after long periods of frequent switching between SWS and REM sleep, giving the subjective impression that the animal was struggling against entering REM sleep. In contrast, bouts of wakefulness were longer with an average duration of 10.2 (±4.8) min and maximum duration of 83.7 (±3.6) min.

### Effects of sleep deprivation/sleep homeostasis

**Figure 3.**
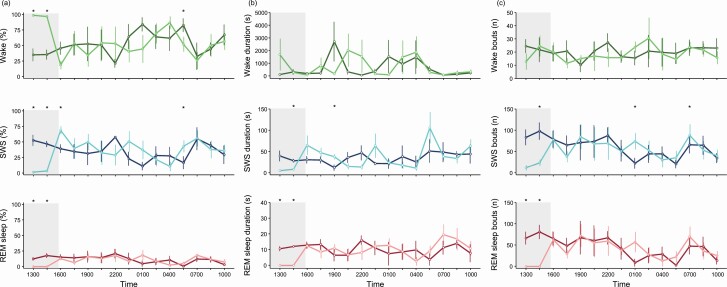
Effect of sleep deprivation on the (a) amount, (b) duration, and (c) number of episodes of wake (top row), SWS (middle row), and REM sleep (bottom row). The first 22.5 h period (dark line) served as an undisturbed baseline; the second 22.5 h period (lighter line) includes the sleep deprivation (grey shading) followed by the recovery period. The black bar along the top reflects the night. Data are presented as *mean* ± *SE* plotted at the start of each 1.5-h time bin. Significant pair-wise differences between the two 22.5 days are indicated by asterisks.

We were successful at reducing sleep, shortening sleep episodes, and reducing the number of sleep bouts (Figure 3). REM sleep did not occur during the sleep deprivation procedure. When allowed to behave freely, the animals engaged in significantly more SWS. The increase in SWS was largely due to an increase in the duration of SWS episodes. The number of SWS bouts was intermittently higher in the middle of the recovery night, and again after lights-on the following morning. Conversely, the amount of REM sleep, and the duration and number of REM sleep episodes, was unaffected by 4-h of enforced wakefulness.

**Figure 4.**
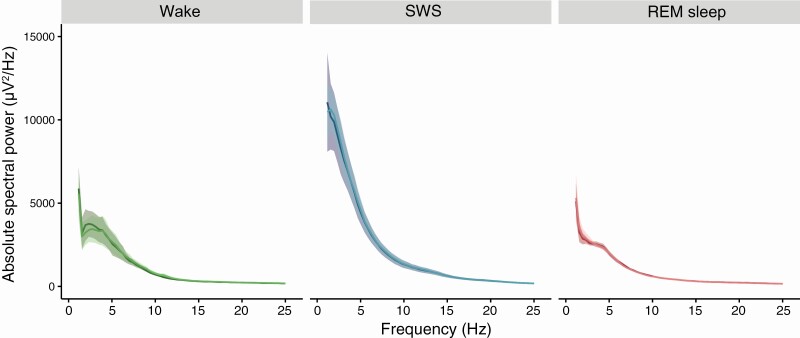
**Mean absolute power spectra (0.78–25 Hz) of the baseline (dark line) and sleep deprivation + recovery (lighter line) for quiet wakefulness, SWS, and REM sleep.** Shaded areas indicate standard error of the mean.

Under baseline and recovery conditions, quiet wakefulness and REM sleep showed similar power spectra; during SWS, low-frequency power spectra was highest (Figure 4, Supplementary Figure S2). We did not detect a theta rhythm during quiet wakefulness or REM sleep. When averaged over the baseline and recovery days, no effect of sleep deprivation was evident on the power spectra for any state. This interpretation changes, however, when looking at finer timescales.

**Figure 5.**
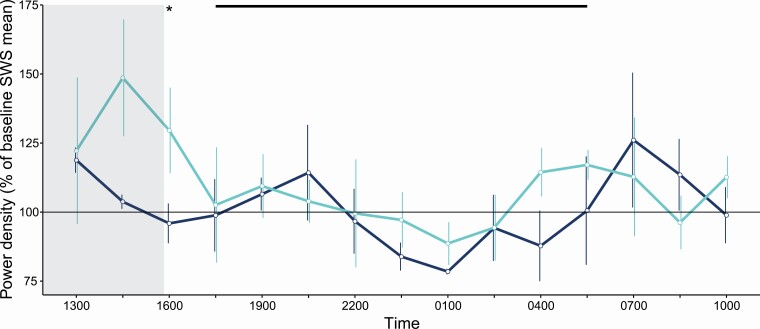
**Effect of sleep deprivation on SWS-related slow-wave activity (0.78–4.30 Hz).** The first 22.5 h period (dark line) served as an undisturbed baseline; the second 22.5 h period (lighter line) includes the sleep deprivation (grey shading) followed by the recovery period. The black bar along the top reflects the night. Data are presented as *mean* ± *SE* plotted at the start of each 1.5-h time bin. The significant pair-wise difference between the two days is indicated by an asterisk.

**Figure 6.**
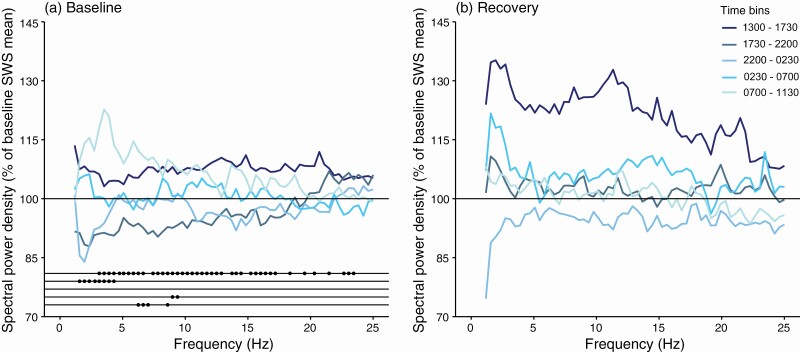
**EEG spectral power density (0.78–25 Hz) during SWS on the (a) baseline and (b) recovery days.** The power density for each 4.5-h time bin is expressed as a percent of the entire baseline SWS mean per frequency bin. Significant differences between the same time bin on the two days are indicated by filled circles on the lines at the bottom of the baseline plot with the top-down order corresponding to that of the time bins in the legend.

SWS-related SWA under baseline conditions showed a daily fluctuation around the mean, with SWA higher during the day. During the sleep deprivation procedure, SWA reached its highest levels, but owing to between-individual variability in the magnitude of the increase, the mean increase was not significant (Figure 5). However, the increase of SWA became significant soon after the deprivation ended when the animals were able to sleep undisturbed. We also explored spectral power density beyond the SWA bandwidth, out to 25 Hz. Spectral power density below 20 Hz appeared higher during the baseline day than during the baseline night (Figure 6). The sleep deprivation caused changes to spectral power density. Specifically, when the animals were allowed to sleep freely, a broad range of frequencies (3–23 Hz) were significantly higher relative to baseline. Power density in the second 4.5-h bin of the recovery period was also enhanced, but only reached significance from 1.6 to 4.3 Hz. Thus, 4 h of sleep deprivation induced a rebound of low-frequency power density that persisted for 9 h.

## Discussion

In this study, we described sleep behavior and physiology of dusky antechinus, and provided the first evidence for SWS homeostasis in any noneutherian mammal. Evidence for REM sleep homeostasis, however, was less clear. Below, we discuss our findings starting with undisturbed (baseline) sleep architecture and timing, and move on to recovery sleep.

### Baseline sleep architecture

Sleep architecture in dusky antechinus was broadly similar to that observed in eutherian and other marsupial mammals. Three vigilance states were observed: wakefulness, SWS, and REM sleep. However, several peculiarities distinguish antechinus. Under baseline conditions, dusky antechinus were awake 11.9 h of the 22.5-h day, and without a preference to sleep during either the day or night. Although there have only been a handful of electrophysiological studies on sleeping marsupials, the common and little water opossums spent much more time asleep than dusky antechinus, upwards of 81% of the 24-h day, of which *circa* 30% was allocated to REM sleep [51–53]. In contrast, the long-nosed potoroo slept a similar amount to dusky antechinus (*c*. 45%), but with much less REM sleep (<6%) [55].

One surprising finding relates to the unexpectedly short duration of SWS and REM sleep episodes in antechinus, an average of 34 s and 10 s, respectively. To the best of our knowledge, these are the shortest sleep bout durations reported for any mammal. Indeed, such low values are more reminiscent of avian sleep than to most mammals [39, 42, 58]. For example, the minute-long REM sleep episodes reported in laboratory mouse strains [59], and two-minute-long REM sleep bouts in the lesser mouse-deer (*Tragulus kanchil*) [60] were still 6–12 times longer than the average antechinus REM sleep episode. The short sleep bouts of antechinus bear some resemblance to the 1–1.5 min oscillation between SWS-like and REM sleep-like states in the central bearded dragon (*Pogona vitticeps*) [61]. Ultimately, additional comparative sleep data on other marsupials would be of value.

While SWS and REM sleep bouts were often short, they could nonetheless be as long as 10 and 3 min, respectively, even if uncommon. Prior to engaging in longer bouts of REM sleep, antechinus would rapidly switch between SWS and REM sleep. Whether this is an idiosyncrasy of antechinus sleep, or a general feature of marsupial biology, is unclear owing to limited data on bout durations in marsupials. It is possible that a perceived increase in the risk of predation stemming from laboratory housing could also have contributed to this phenomenon [62]. That said, we tried to reduce any “lab-effect” by having nearly two months of acclimation before experimental recordings began, maintaining a natural day–night cycle, and providing environmental enrichments (e.g. habitat complexity, natural sounds and substrate, and live food).

Another interesting finding relates to the presence of shivering-like activity during SWS. The movement that generated this accelerometry signal was too small to be captured by our video cameras, so we cannot see the behavior. Moreover, the signal was not evident in the electromyogram, indicating that the neck muscle does not shiver. Other muscles must be responsible. Nonetheless, it remains compelling that shivering occurred during SWS, but not REM sleep. Eutherian mammals and birds can thermoregulate (i.e. pant, sweat, shiver) during SWS, but these behaviors cease during REM sleep [63–65]. This pattern suggests that the shivering-like activity we observed in antechinus was indeed shivering.

SWS-related thalamocortical spindles and REM sleep-related hippocampal theta are common features of sleep in eutherian mammals. Sleep spindles have also been observed across diverse marsupials, including the common opossum (8–11 Hz) [51], North American opossum (10–14 Hz) [56], white-eared opossum (*Didelphis albiventris*) (10–16 Hz) [66], little water opossum [53], and long-nosed potoroo [67]. Sleep spindles (7–13 Hz) were also found in dusky antechinus, notably, but not exclusively, at SWS-REM sleep transitions. A hippocampal theta rhythm was reported in the brushtail possum [54] and only during wakefulness in the monotreme, the short-beaked echidna (*Tachyglossus aculeatus*) [68]. That said, we did not detect a theta rhythm in dusky antechinus. Future studies should employ depth local field potential recordings to determine whether antechinus generate a hippocampal theta rhythm.

In mammals, sleep is controlled by a homeostatic process, which regulates the depth (or intensity) of SWS, and by the circadian clock, which regulates sleep timing [7, 69]. Although we studied only four individuals, we nonetheless observed surprising intra-specific variation in the timing of sleep. One animal showed a preference to sleep during the night, another during the day, and two others that oscillated with varying ultradian rhythms. This seems to indicate that sleep in antechinus is weakly regulated by time-of-day. Instead, the variability observed in the timing of sleep may be adaptive given the unusual life-history strategy of all antechinus, which is characterized by a single, short, and intense breeding season after which all males die. Cathemeral sleep–wake patterns may provide the behavioral flexibility needed to expand their temporal niche during the breeding season to avoid intra-sexual competition for access to females and ensure paternity.

This cathemeral organization of behavior is likely the cause for modest dynamics of SWS SWA under baseline conditions. Mammals that show a marked preference to consolidate wakefulness either during the day (chipmunk and human) or night (rat and hamster), build up a sleep debt that manifests as declining SWA with time asleep. Conversely, mammals that sleep more indiscriminately (cat, guinea pig, and dusky antechinus), do not build up a substantial sleep debt because wakefulness is curtailed by frequent naps, and so SWA shows less variation across the 24-h day. The temporal organization of REM sleep in dusky antechinus was uniform across the recording period, resembling that of other arrhythmic species [25, 26, 32, 33]. This contrasts with species that consolidate their sleep, wherein the amount of REM sleep increases toward the end of the major sleep phase [21–23].

### Recovery sleep architecture

Following an effective 4-h sleep deprivation, there was more SWS, of greater continuity, and enhanced SWA. SWA also showed a clearer decline across the recovery period. These responses to extended wakefulness have been reported in eutherian mammals [7, 22, 26]. Our results thus demonstrate that SWS homeostatic processes manifest similarly in eutherian and marsupial mammals.

Interestingly, although REM sleep was eliminated during the sleep deprivation, dusky antechinus did not show any rebound of REM sleep over the recovery period. The amount of REM sleep was not higher, nor were episodes of REM sleep longer or more numerous. Similarly, in most studies on rats and cats, sleep loss of 12 h or less was not followed by a clear rebound in REM sleep [21, 30, 33]. Conversely, REM sleep homeostasis has been demonstrated in several eutherian mammals and birds, typically when deprived of sleep for longer (8**–**24 h) periods of time. Thus, it is unclear whether REM sleep would have increased following a longer period of sleep loss in antechinus. That said, Australian magpies [42, 43], European starlings [40], ferrets [34], tree shrews [35], and Siberian chipmunks [23], did not show any REM sleep rebound following 6–24 h of extended wakefulness, nor did northern fur seals (*Callorhinus ursinus*) deprived of REM sleep for as long as 2 weeks [47]. In contrast, a series of studies on Djungarian hamsters (*Phodopus sungorus*) reported contradictory results over the existence of REM sleep homeostasis after shorter periods of sleep loss [70, 71]. In the guinea pig, the amount of REM sleep was influenced by the circadian time at which the deprivation occurred [26]. In Syrian hamsters, REM sleep was higher only after 24 h of sleep deprivation by forced locomotion, but not gentle handling [22]. Similarly, in rats and mice, immobilization-induced wakefulness, but not unescapable foot-shock, caused a REM sleep rebound [72, 73]. As such, the reasons for the absence of a REM sleep rebound can be diverse. Future studies should investigate dose-dependent responses of sleep loss in marsupials, delivered at different times of the 24-h day, and using varied techniques.

In conclusion, we showed that sleep architecture in dusky antechinus was broadly similar to that observed in eutherian and other marsupial mammals, but with several peculiarities. Dusky antechinus showed unexpectedly short, bird-like, episodes of SWS and REM sleep, and surprising intra-specific variation in the timing, but not amount, of sleep. Following sleep deprivation, dusky antechinus recovered lost SWS by sleeping longer, with greater continuity, and enhanced SWA. This provides the first evidence of SWS homeostasis in marsupial mammals. Dusky antechinus did not show a rebound of REM sleep over the recovery period; however, additional study, using longer periods of sleep loss, is needed to fully understand the reason(s) for the apparent lack of REM sleep homeostasis in antechinus.

## Supplementary Material

zsac114_suppl_Supplementary_Figures
