# Supplementary material for: Sleep architecture and regulation of male dusky antechinus, an Australian marsupial
Source: Sleep. 2022 May 14;45(8):zsac114. doi: 10.1093/sleep/zsac114 (PMC9366648; doi:10.1093/sleep/zsac114)
Supplement: zsac114_suppl_Supplementary_Figures [file zsac114_suppl_supplementary_figures.pdf]

SUPPLEMENTARY FIGURES

Sleep architecture and regulation of male dusky antechinus, an Australian marsupial

Erika Zaid[1*], Alexei L. Vyssotski[2] and John A. Lesku[1*]

[1]School of Agriculture, Biomedicine and Environment, La Trobe University, Melbourne, Australia

[2]Institute of Neuroinformatics, University of Zurich/ETH Zurich, Zurich, Switzerland

*Correspondence: erikazaid@gmail.com; j.lesku@latrobe.edu.au

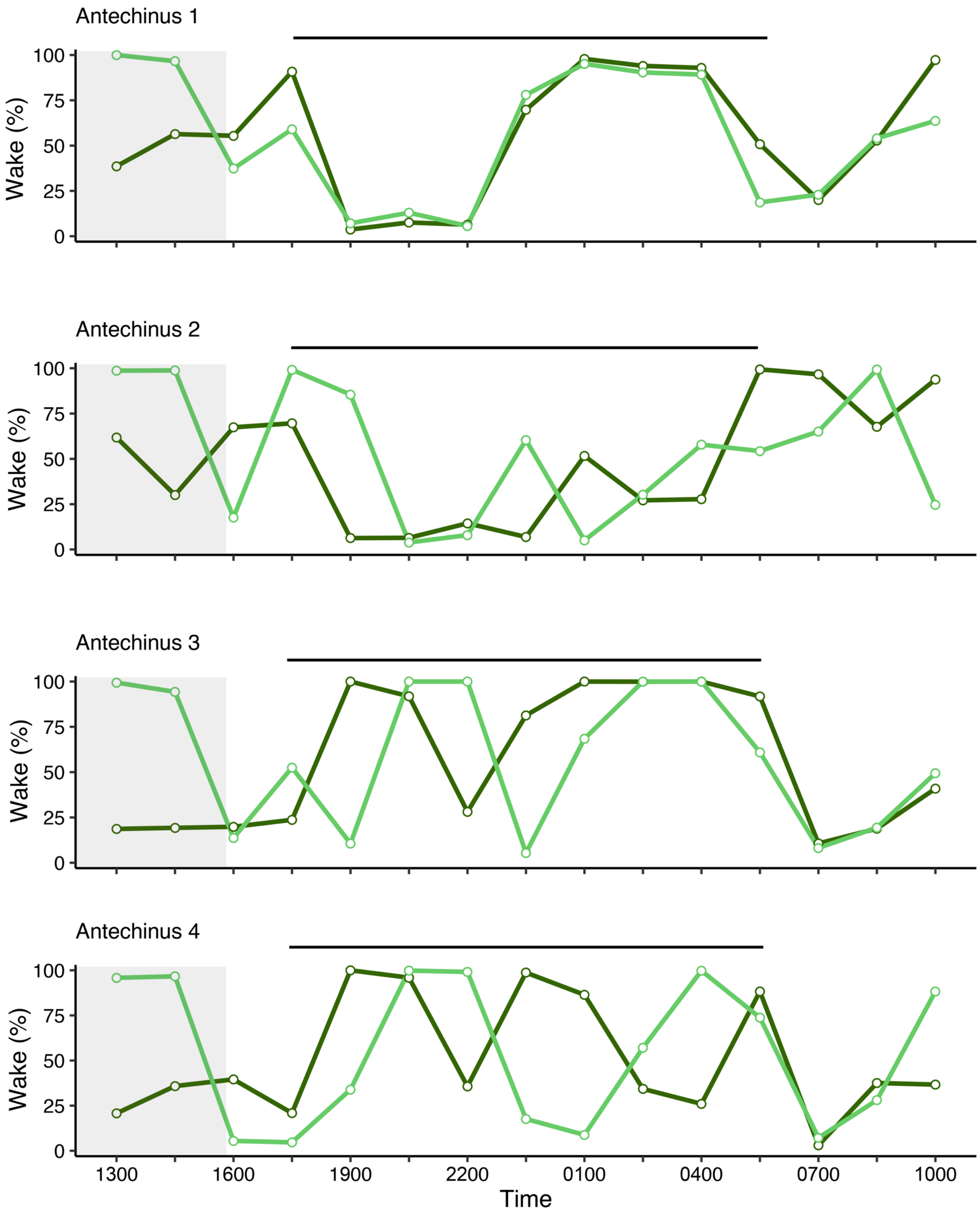

Antechinus 1
Antechinus 2
Antechinus 3
Antechinus 4
Wake (%)
0
25
50
75
100
1300
1600
1900
2200
0100
0400
0700
1000
Time

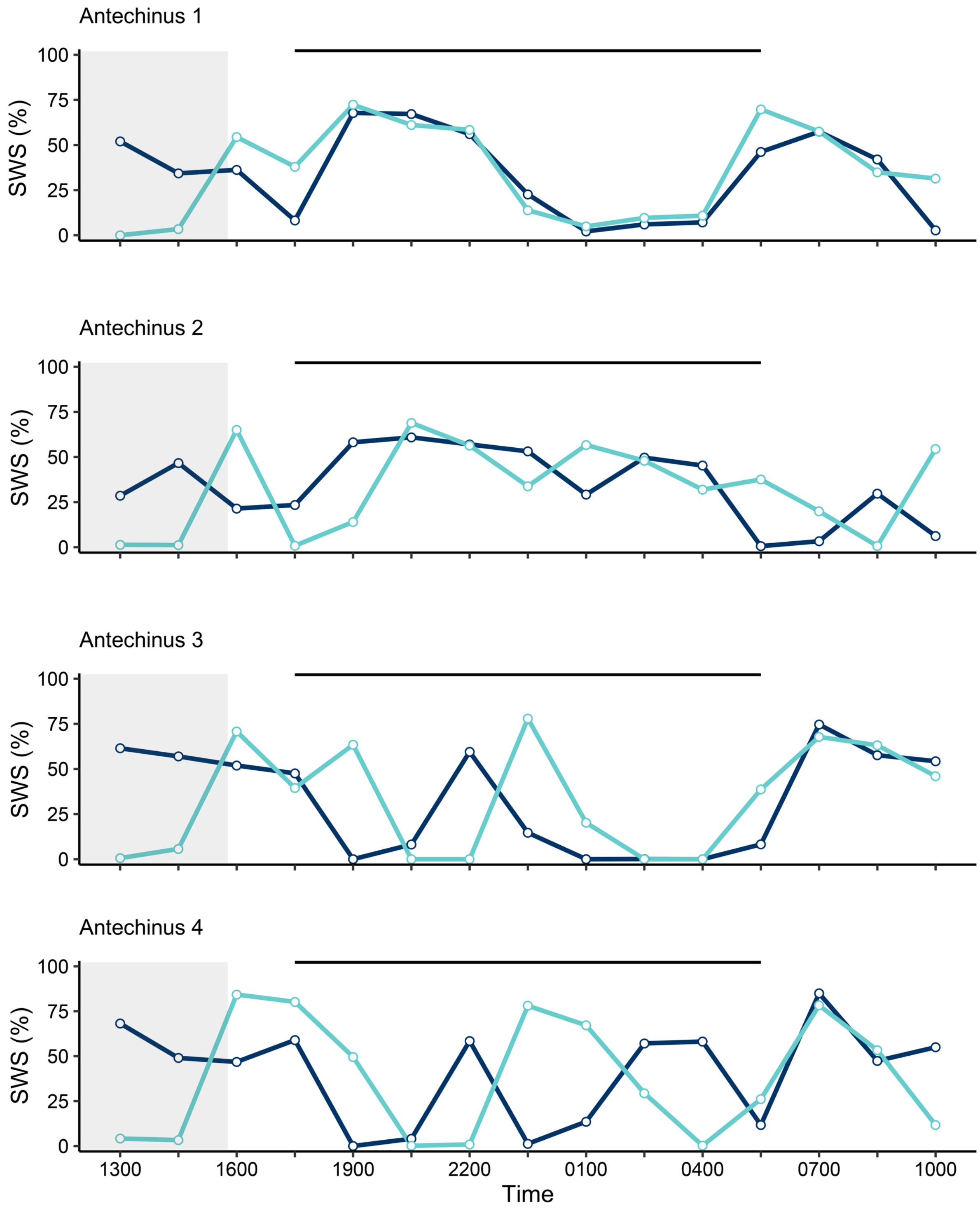

Antechinus 1
Antechinus 2
Antechinus 3
Antechinus 4
SWS (%)
100
75
50
25
0
1300
1600
1900
2200
0100
0400
0700
1000
Time

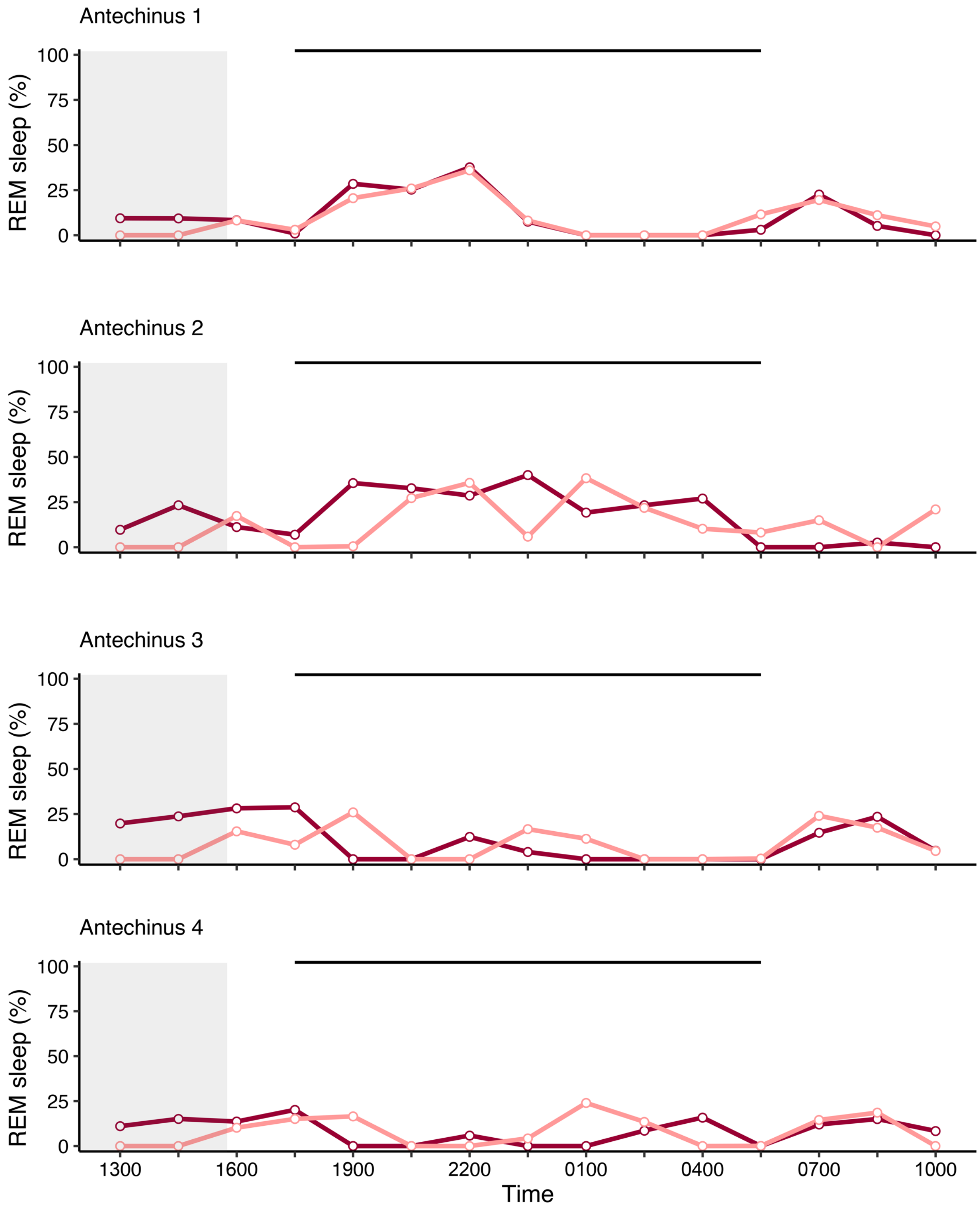

Supplementary Figure 1. Individual variation on the amount of wake (green), SWS (blue), and REM sleep (red). The first 22.5 h period (dark line) served as an undisturbed baseline; the second 22.5 h period (lighter line) includes the sleep deprivation (grey shading) followed by the recovery period. The black bar along the top reflects the night. Data are plotted at the start of each 1.5-h time bin.

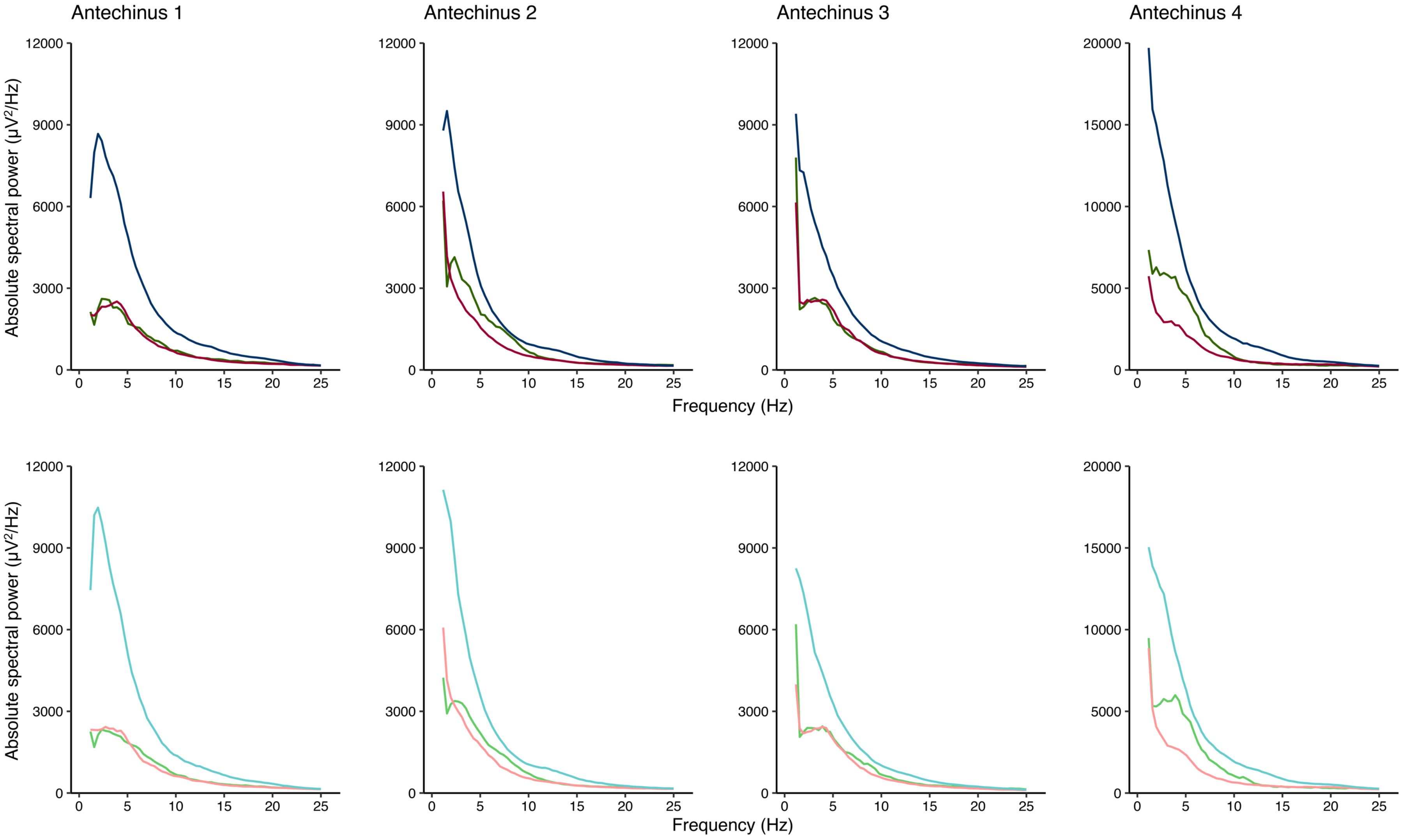

Supplementary Figure 2. Individual variation of absolute power spectra (0.78 – 25 Hz) during quiet wakefulness (green), SWS (blue), and REM sleep (red) during the baseline (top row) and sleep deprivation + recovery (bottom row). In all animals, power was calculated using the electrodes over the right hemisphere.
